# Supplementary material for: Effect of Temperature on Passive Film Characteristics of LPBF (Laser Powder-Bed Fusion) Processing on UNS-S31603
Source: Materials (Basel). 2024 Jul 11;17(14):3420. doi: 10.3390/ma17143420 (PMC11278099; doi:10.3390/ma17143420)
Supplement: Supplementary file 1 [file materials-17-03420-s001.zip › materials-3078287-supplementary.pdf]

Supplementary Materials

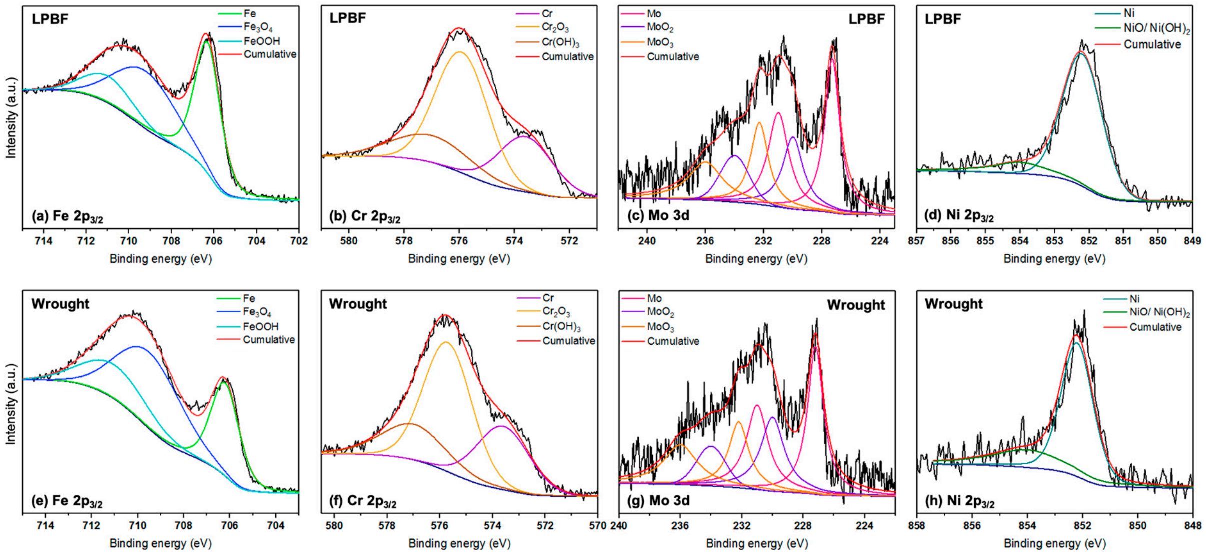

Figure S1. Individual high resolution XPS spectra of the Fe $2p_{3/2}$, Cr $2p_{3/2}$, Mo 3d and Ni $2p_{3/2}$ peaks obtained for the (a–d) LPBF and (e–h) Wrought SS316L samples after 1 day of exposure in the buffered 3.5 wt% NaCl solution at 25°C.

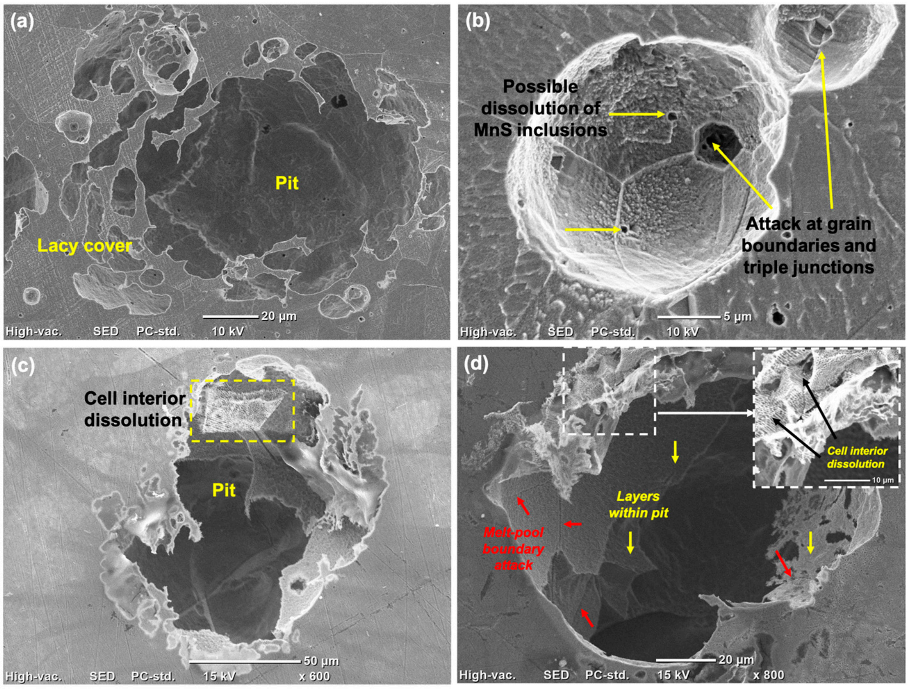

Figure S2. Pits formed on (a,b) Wrought and (c,d) LPBF SS316L samples after CPP in buffered 3.5 wt% NaCl solution at 25°C.
